# 533. Use of Different Communication Types for Telehealth for COVID-19 During Implementation of a State Telehealth Test to Treat Program in Minnesota

**DOI:** 10.1093/ofid/ofad500.602

**Published:** 2023-11-27

**Authors:** Sarah Lim, Paige Dubelko, Walter James, Erin McLachlan, David Tsay, Ruth Lynfield

**Affiliations:** Minnesota Department of Health, St. Paul, Minnesota; Cue Health, San Diego, California; Minnesota Department of Health, St. Paul, Minnesota; Minnesota Department of Health, St. Paul, Minnesota; Cue Health, San Diego, California; Minnesota Department of Health, St. Paul, Minnesota

## Abstract

**Background:**

Telehealth programs for COVID-19 have expanded and may use multiple types of communication, such as mobile apps, web-based or telephone. Data on use of these communications are lacking, particularly in different age groups. We assessed use of three different communication types during implementation of a test to treat program for COVID-19 in Minnesota.

**Methods:**

Minnesota launched its telehealth program in two phases: Phase 1 (mobile app only) during initial rollout from 12/15/22 – 1/5/23 and Phase 2 (addition of web and phone options) from 1/6/23 – 4/30/23. Data on users of the program were provided by the vendor (Cue Health), including communication type (app, web, phone), number of attempts, antiviral prescription, age, and sex.

**Results:**

Data were available for 2093 patients who completed the intake process between 12/15/22 and 4/30/23, of whom 1928 (92%) completed the consultation process and 1808 (86%) received an antiviral. 60% of users were female. 250 patients (12%) completed the intake more than once on the same day, which may reflect multiple attempts due to technical barriers. During the study period, patients aged 35-44 and ≥ 65 years made up the largest proportion of users by age group (each had 464 patients, each 22% of total, Figure 1). 37% of users accessed the program via the phone and 37% via the app, with 26% using the website. Communication preferences varied by age, with increased use of the phone option in older age groups (54% of ≥ 65 vs 30% of 18-34 years, Figure 2). In comparison to users who made a single attempt, users who made multiple attempts were more likely to be younger (48 vs 51 years, p=0.02), and less likely to be prescribed an antiviral (80% vs 91%, p< .001). Users with multiple attempts were more likely to have used the web option for their first attempt (47% vs 24%, p< .001). Those with multiple attempts generally used the same communication type for all attempts (204/250); of those who switched, the commonest switch was to the phone (39/46).

**Figure:**
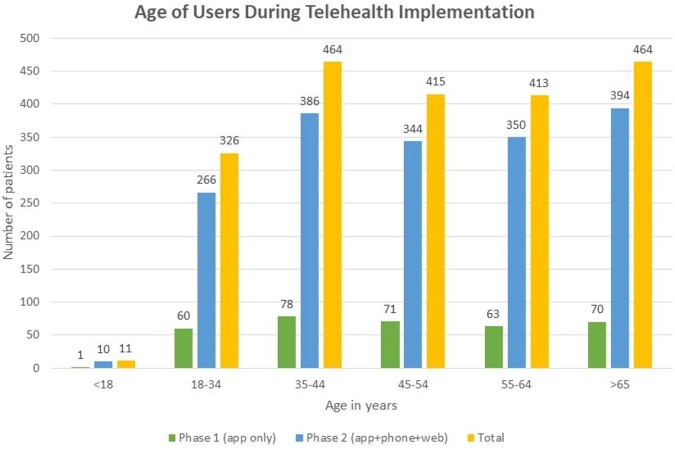

**Figure:**
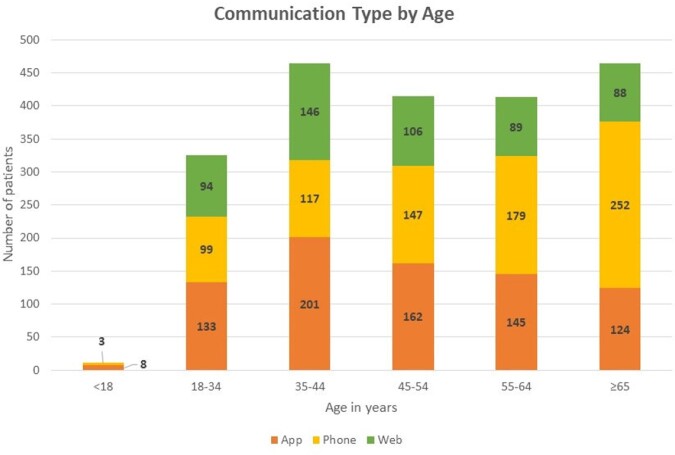

**Conclusion:**

Of the three communication types, the phone and app options were most popular, with a trend towards more phone use in older age groups. A phone option should be considered in future telehealth initiatives, including as a backup for those with technical barriers to app or web-based communication.

**Disclosures:**

**David Tsay, MD PhD**, Cue Health: Chief Medical Officer|Cue Health: Stocks/Bonds

